# Wire Arc Additive Manufacturing of Zinc as a Degradable Metallic Biomaterial

**DOI:** 10.3390/jfb13040212

**Published:** 2022-11-01

**Authors:** Rishabh Soni, Suyog Jhavar, Suhela Tyeb, Saurabh Kumar Gupta, Satyam Suwas, Kaushik Chatterjee

**Affiliations:** 1 Department of Materials Engineering, Indian Institute of Science, Sir C. V. Raman Avenue, Bangalore 560012, India; 2 School of Mechanical Engineering, VIT-AP University, Inavolu, Beside AP Secretariat Amaravati, Amaravati 522237, India

**Keywords:** additive manufacturing, resorbable implants, metallic biomaterials, Zn implants

## Abstract

Wire arc additive manufacturing (WAAM) offers a high rate of material deposition among various additive manufacturing techniques with wire as feedstock material but has not been established for zinc alloys. Zn alloys can be used as degradable biomaterials, in contrast to conventional permanent metallic biomaterials. In this work, commercially pure Zn was processed by WAAM to obtain near-dense parts, and the properties obtained through WAAM-processed Zn were compared with wrought (WR) Zn samples. The microstructure and hardness values of the WAAM (41 ± 1 HV0.3) components were found to be similar to those of the WR (35 ± 2 HV0.3) components. Bulk X-ray diffraction texture measurements suggested that WAAM builds exhibit a heavily textured microstructure compared to the WR counterparts, with peak intensities around <3 3–6 2> or <0 0 0 2> in the directions parallel to the build direction (BD). The corrosion rates in simulated body fluid (SBF) were similar for WAAM (0.45 mmpy) and WR (0.3 mmpy) samples. The weight loss measurements in SBF were found to be marginally higher in the WAAM samples compared to the WR counterparts for a duration of up to 21 days. MC3T3-E1 preosteoblasts were found to be healthy and proliferating in the culture medium containing the degradation products from WAAM-Zn in a manner similar to WR-Zn. This work establishes the feasibility of processing Zn by WAAM for use in bioresorbable metallic implants.

## 1. Introduction

The fabrication of metallic implants for clinical applications majorly employs non-degradable alloys such as stainless steel, titanium alloys, cobalt chrome alloys, etc. However, in transient applications, such as fracture fixation, these permanent implants necessitate a second removal surgery and risk serving as sites for infections [1,2]. Biodegradable metals and alloys, which eliminate the need for a second surgery and hence reduce the risk of infection, are being actively researched as promising alternatives for such applications [3]. Fe-, Mg-, and Zn-based alloys are the most widely researched biodegradable materials [4]. However, Mg-based implants exhibit higher degradation rates and are resorbed before the implant fully serves its intended purpose [5]. On the other hand, Fe and its alloys, despite showing good mechanical properties, have lower degradation rates in the physiological environment and hinder the healing of bone tissue [6,7]. Unlike Mg or Fe, the biodegradation rate of Zn better matches the rate of bone tissue regeneration [8]. In addition, the degradation of Zn does not generate H_2_ gas, which can damage the regenerating bone and trigger chronic inflammation, as seen for Mg alloys [9,10].

Additive manufacturing (AM) technologies have been rapidly emerging in recent years for various industries [11]. AM essentially requires a heat source to melt the feed material and deposit it in a layer-by-layer fashion into a pre-modeled component. AM techniques are broadly divided into two categories, namely powder bed fusion (PBF) and directed energy deposition (DED) [12]. AM offers many benefits over conventional manufacturing techniques and is particularly attractive for preparing bioimplants with complex geometry to meet the individual needs of patients [13].

Wire arc additive manufacturing (WAAM) is one such DED-AM technique that utilizes feedstock in the form of a wire, thus eliminating the complications associated with powder deposition. In WAAM, an arc-generated plasma is used to melt the wire to deposit it on the substrate mounted on the manipulator. In contrast to PBF systems, WAAM enables faster production owing to fewer post fabrication processes. Moreover, controlling the heat source is easier in the case of a plasma arc compared to laser or electron beam sources. This difference also helps in controlling the depth of penetration of the heat source compared to powder bed systems [14]. Wire-feed-based AM processes are user-friendly and less hazardous to health compared to powder-feed-based processes. Using wire as a material feed also mitigates the problems of contamination and oxidation associated with powder-based techniques, as the surface-to-volume ratio in wires is much less than in powders [14]. Further, since WAAM does not involve the recycling and reuse of a powder that adds to the contamination, it does not deteriorate the quality of fabrication.

The AM of Zn-based alloys is challenging as Zn alloys have low melting (420 °C) and boiling (907 °C) points and hence are prone to oxidation and porosity formation with high-energy laser or electron beams [15]. Few attempts are reported in the literature on the AM of Zn and its alloys through laser-based PBF techniques [16,17,18,19,20,21,22,23,24,25]. Demir et al. demonstrated the feasibility of preparing Zn parts with >99% density with minimal defects by SLM. The high cooling rate of SLM resulted in fine grains with enhanced strength compared to conventionally manufactured parts [16]. Elongated grains along the build direction were reported in additively manufactured parts that imparted anisotropy to its mechanical properties [19,20,24,25]. Li et al. have reported the suitability of porous Zn scaffolds prepared by SLM for biodegradable bone implants [21,22,23]. They reported that the mechanical properties of the scaffolds were found to be similar to those of cancellous bone. Furthermore, the biodegradation products of these Zn scaffolds were observed to be nontoxic to MG-63 cells [23]. Other reports have also described the favorable biological responses of additively manufactured Zn components [21,22,24,25]. However, despite its utility, there are no reports on the fabrication of Zn alloys by WAAM for the fabrication of degradable implants. The goal of this work was to establish the feasibility of preparing dense parts of commercially pure Zn by WAAM and to investigate its properties as a potential degradable biomaterial.

## 2. Materials and Methods

### 2.1. Materials

Commercially pure Zn (>99.9%) from Vedanta Ltd. (Mumbai, India) was used as the wrought material. Cast Zn blocks were used after recrystallization at 300 °C for microstructural homogenization. Commercial high-quality Zn wires from the same company (>99.9% Zn) with a diameter of 1.22 mm served as the feedstock for WAAM. The deposition of the feedstock was performed on substrates of the same composition. The thickness of the substrates was fixed at 12 mm. The substrates were polished with 320-grit sandpaper and cleaned with acetone to scrub off the unwanted debris and oxide particles.

The following reagents were procured for the cell studies: MC3T3-E1 cells (ATCC), phosphate-buffered saline (PBS, pH 7.4), α-minimal essential medium (α-MEM, Gibco, Life Technologies), fetal bovine serum (FBS, EU-approved South American origin was purchased from Gibco, Bangalore, India), Anti-Anti (antibiotic-antimycotic, Thermo Fischer Scientific, Powai, India), fluorescein isothiocyanate (FITC)-conjugated phalloidin (Thermo Fischer Scientific, Powai, India), and 4,6-diamidino-2-phenylindole (DAPI, Invitrogen, Thermo Fischer Scientific, Powai, India).

### 2.2. Additive Manufacturing of Zn

The WAAM unit for this work was described in an earlier study [26]. Plasma arc generation was carried out in an inert atmosphere of argon shielding gas. The gas flow rate for argon was kept constant at 3 L/min for shielding and arc generation. The distance between the arcing nozzle and the deposition substrate was held constant at 5 mm. The wire was fed at a fixed angle of 45° into the arcing zone with a constant wire feed rate of 160 mm/min. The arc power and travel speed of the torch were kept constant at 400 W and 120 mm/min for optimal deposition.

#### Microstructural Characterization and Texture Studies

Small pieces of WR-Zn and WAAM-Zn were cut out from the bulk and polished using sandpaper up to 3000-grit. A mirror-like surface finish was achieved after electropolishing with an electrolyte consisting of orthophosphoric acid and ethanol in a 3:5 ratio. Electropolishing was performed at an applied voltage of 20 V for 35 s and maintained at 0–5 °C. Polished surfaces were etched for 10 s using 2% nitric acid to analyze the grain size and morphology under an optical microscope (OM, Zeiss, Oberkochen, Germany) and a scanning electron microscope (SEM, TESCAN MIRA 3, Brno, Czech Republic). The time of exposure to the etchant was 6 s for WAAM-Zn, whereas it was 10 s for WR-Zn. Grain size calculations were performed with ImageJ software using the line intercept method.

X-ray diffraction measurement was performed on the BD-SD plane of WAAM-Zn and the RD-TD plane of WR-Zn samples in the 2θ range of 30 to 80° with a Cu Kα wavelength in a Rigaku SmartLab XRD. The bulk textures of the WR-Zn and WAAM-Zn samples were measured with a Cu Kα wavelength in an X-ray texture goniometer (Rigaku SmartLab XRD, Tokyo, Japan) based on Schulz reflection geometry operating at 40 kV and 40 mA on the BD parallel surface of WAAM and the ND surface of WR. Six incomplete pole figures (10.0, 00.2, 10.1, 10.2, 11.0, and 10.3) were measured initially and were utilized to calculate the orientation distribution function (ODF) using ATEX software (version 3.x, Jean-Jacques Fundenberger and Benoit Beausir, Metz, France) [27]. Inverse pole figures were generated for a texture analysis. The samples for texture measurement were prepared by following standard metallographic techniques.

### 2.3. Corrosion Studies

A standard three-electrode potentiostat (C. H. Instruments, CHI604E, Texas, TX, USA) with a standard calomel reference electrode and a platinum (Pt) counter electrode was used to study the electrochemical responses of the samples. Independently, mounted samples with exposed areas of 5 × 5 mm^2^ of each WAAM-Zn and WR-Zn sample were prepared and polished up to 3000-grit. The open-circuit potential of the samples was first stabilized by keeping the samples in simulated body fluid (SBF, prepared as described in [28]) for 1 h with no voltage differences. Samples were then exposed to 10 mL of SBF at room temperature with an applied voltage varying from −2 V to 0.5 V at a constant voltage sweep rate of 10^−3^ s^−1^.

### 2.4. Microhardness Tests

Microhardness tests were performed on the polished surfaces of the samples using a Vickers microhardness tester (Future-Tech FM-800, Kanagawa, Japan) with a diamond indenter. The indenter load and dwell time were fixed at 300 gf with a 10 s dwell time. At least 15 measurements were taken at different locations for a given sample.

### 2.5. Degradation Behavior

The samples were cut in the dimensions of 5 × 5 × 2 mm^3^ for the biodegradability tests. Each sample was immersed in 10 mL of SBF solution and kept in an incubator shaker operating at 100 rpm and 37 °C for up to 3 weeks. The weights of the samples were measured 7, 14, and 21 days after the start of the incubation. The samples were washed and dried prior to weighing. The SBF medium was regularly replenished every third day.

### 2.6. Cell Compatibility Studies

The cytocompatibility of WAAM-Zn was determined by the indirect method with MC3T3-E1 cells (ATCC) and compared to that of WR-Zn. Cuboid-shaped samples (7 × 7 × 2 mm^3^) were cut and polished. The samples were sterilized by incubating in ethanol, followed by exposure to UV for 1 h. The samples were further washed with sterile phosphate-buffered saline (PBS, pH 7.4) with an antibiotic (1%) three times to remove any residual ethanol on the sample.

The effect of any leaching from the alloy on the cellular activity was observed by preparing the conditioned medium. Briefly, the sterilized samples were incubated with complete medium (α-minimum essential medium (α-MEM) + 10% fetal bovine serum (FBS) + 1% antibiotic) for 24 h at 37 °C and 5% CO_2_. The volume of medium to sample was kept constant at 15 µL mm^−2^. Following incubation, the metal samples were removed, and the conditioned medium was centrifuged at 5000 rpm for 20 min to remove any debris.

MC3T3-E1 cells were cultured in complete medium (α-MEM + 10% FBS + 1% antibiotic) and passaged at 70% confluency, as reported recently [29]. Cells were seeded at a density of 3 × 10^3^ cells per well in a 48-well plate and allowed to attach for 24 h by incubating at 37 °C and 5% CO_2_. Following incubation, the medium was replaced by 300 µL of conditioned medium and further incubated for 48 h. Cells that were cultured in fresh medium served as the positive control. Cell viability was quantitively evaluated by the Resazurin assay.

After incubation, the medium was removed, and the cells were washed with PBS and incubated with Resazurin solution (200 µL, 0.015 mg/mL) for 3 h at 37 °C and 5% CO_2_. The dye was reduced to a pink-colored fluorescent resorufin solution by viable cells. The fluorescence was measured by excitation at 530 nm and emission at 590 nm using a plate reader (Biotek Gen 5, Santa Clara, CA, USA). The Resazurin assay was also performed before the addition of the conditioned medium to serve as a reference. The results are reported as fold changes in the fluorescence intensity of the cells before and after treatment with the conditioned medium. Similarly, for the positive control, the results are reported as a fold change in fluorescence intensity before and after incubation in a fresh culture medium.

Following our recently reported procedure [29], the viability of the cells was also qualitatively evaluated by staining the cells with Calcein AM (Thermo Fischer Scientific, Powai, India) and Ethidium Homodimer dye (Thermo Fischer Scientific, Powai, India) and imaging with an inverted epi-fluorescence microscope (Olympus, Tokyo, Japan).

To visualize the cytoskeletal integrity, cells were fixed in 4% paraformaldehyde in a PBS solution (pH 7.4) at room temperature. After washing with PBS, cells were permeabilized by incubating with a 0.1% Triton X-100 solution (Sigma, Darmstadt, Germany) for 10 min. The cells were then washed with PBS and incubated with 10 µg mL^−1^ fluorescein isothiocyanate (FITC)-conjugated phalloidin (Thermo Fischer Scientific, Powai, India) for 1 h, followed by incubation with a 1 µg mL^−1^ 4,6-diamidino-2-phenylindole (DAPI, Invitrogen, Thermo Fischer Scientific, Powai, India) solution for 5 min to stain the nuclei and F-actin. The stained cells were observed under an inverted epi-fluorescence microscope (Olympus, Tokyo, Japan).

All quantitative assays were performed in triplicate, and a single-factor ANOVA was performed to obtain the significance between the two different alloy types. The significance level was taken as *p = 0.05*, i.e., p < 0.05 (*), p < 0.01 (**), p < 0.0001 (***), and p > 0.05 (ns—nonsignificant).

## 3. Results and Discussion

**Figure 1:**
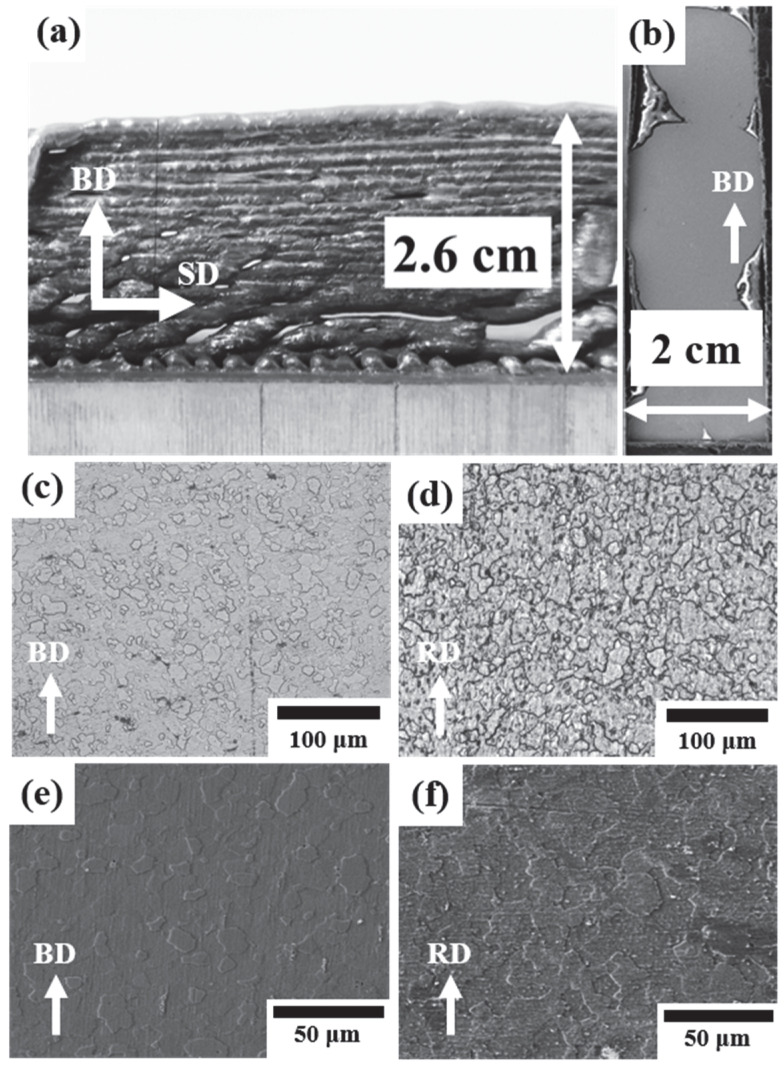
(**a**) Zn part of 16 deposited layers built by WAAM; (**b**) SEM image of 8 deposited Zn layers, showing dense parts with few defects; (**c**,**d**) optical micrographs of (**c**) WAAM-Zn and (**d**) WR-Zn; (**e**,**f**) SEM images of (**e**) WAAM-Zn and (**f**) WR-Zn (BD = building direction, SD = scanning direction, RD = rolling direction).

Commercially pure zinc wire was used as the feedstock to prepare 3D-printed parts through WAAM. The 3D-printed parts were compared with wrought material of cast Zn blocks recrystallized at 300 °C for microstructural homogenization. Owing to its low melting point (420 °C) and boiling point (907 °C), which results in high evaporation of the metal, Zinc requires a narrow range of AM parameters [16]. Mg and its alloys also present similar challenges due to the similarities in the properties of these two metals [30]. The energy input of 60–135 J/mm^3^ was reported to obtain the optimum build quality in SLM for Zn and its alloys [19]. The parameters for the WAAM of pure Zn were optimized for preparing dense samples (Table 1). A representative printed part for 16 deposited layers is shown in Figure 1a. Figure 1b is a scanning electron micrograph that reveals that the build components had densely deposited layers with minimal evaporated voids.

**Figure 2:**
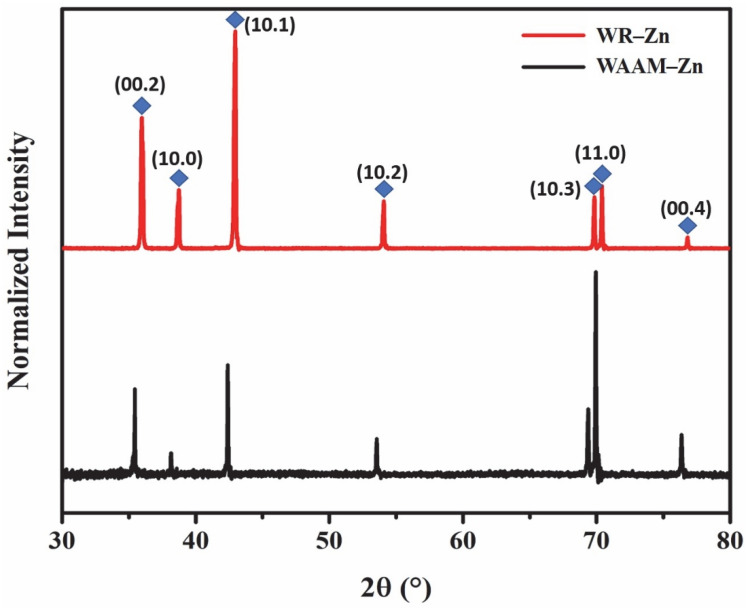
X-Ray diffraction patterns for WAAM-Zn and WR-Zn.

The samples, hereafter referred to as WAAM-Zn, were etched, and the microstructure was characterized with optical and scanning electron microscopes and compared with that of wrought commercially pure Zn (WR-Zn) of a similar composition. The exposure time to etch the microstructure was lower in the case of WAAM-Zn compared to the WR-Zn samples. This could be due to the higher reactivity of the WAAM-Zn compared to WR-Zn. The micrographs in Figure 1 reveal that the mean grain sizes were 14 ± 5 μm for WAAM-Zn and 11 ± 4 μm for WR-Zn. The WAAM-Zn samples were observed to have marginally larger grains on average due to the repetitive heating cycle of the printed layers. Similar phenomena have been reported in multilayer walls and blocks built by AM [31]. X-ray diffraction measurements were performed, and the normalized intensities are plotted in Figure 2. The data reveal the presence of the Zn phase alone in both conditions. The {1 1–2 0} peak in the X-ray diffraction pattern of the WAAM-Zn indicates the presence of a crystallographic texture in WAAM-Zn.

**Figure 3:**
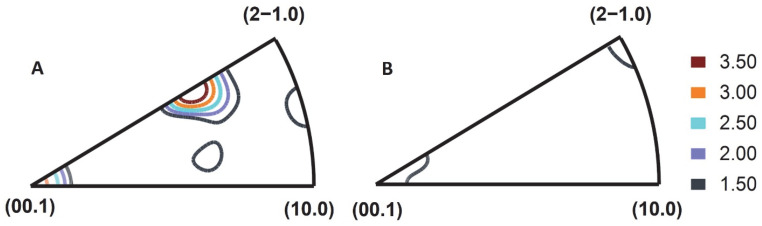
Inverse pole figures for (**A**) BD-WAAM-Zn and (**B**) ND-WR-Zn.

The build-direction inverse pole figure (BD-IPF) in WAAM-Zn and the normal-direction inverse pole figure (ND-IPF) in WR-Zn are plotted in Figure 3. Well-defined texture components were observed in the WAAM-Zn samples, whereas a weak texture was noticed in WR-Zn samples. Due to the rapid cooling and solidification in AM, a similar strong texture is imparted in the 3D-printed components [32]. The analysis of the BD-IPF for the WAAM-Zn condition suggests that the build direction is closely parallel to either the <3 3–6 2> or <0 0 0 2> directions of the grains, whereas in WR-Zn the ND is closely parallel to the <1 1–2 0> or <0 0 0 2> directions of the grains. Notably, the texture of a biomaterial can profoundly influence its performance [33,34].

The Vickers microhardness value were found to be 35 ± 2 HV0.3 at the load of 300 gf for WAAM-Zn samples, whereas it was higher in WR-Zn, at 41 ± 1 HV0.3, as tabulated in Table 2. The lower hardness values in WAAM samples agree well with the marginally larger grain size values of around 14 μm, which result in a smaller grain boundary area and less resistance to indentation [35]. Peng et al. reported similar hardness values of 41 HV for Zn prepared by SLM with 99.5% density [18].

**Figure 4:**
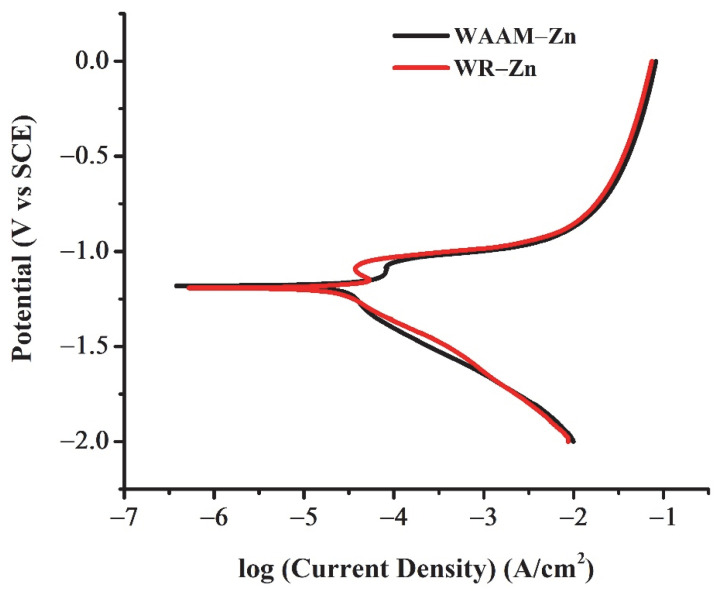
Electrochemical behavior of WAAM and WR-Zn.

The corrosion behavior of the WAAM-Zn and WR-Zn samples was analyzed by Tafel extrapolation. The potentiodynamic polarization curves are shown in Figure 4. The values determined by the Tafel extrapolation technique are compiled in Table 2. The electrochemical activity of a metal depends on the characteristics of the exposed surface area of the metal. Grain size or grain boundary area, surface roughness, and chemical inhomogeneity are some of the key factors that influence corrosion behavior [36]. The similar values of E_corr_ for the WAAM-Zn and WR-Zn samples (−1.18 V and −1.13 V, respectively) arose due to the similar grain sizes and morphologies of these samples. The corrosion current density, I_corr_, is a measure of the rate of Zn^2+^ release into solution. The values of I_corr_ were also similar for both samples. The corrosion rates were calculated from the E_corr_ and I_corr_ values and are tabulated in Table 3. The similar corrosion rates of WAAM-Zn and WR-Zn are in agreement with the similar grain morphologies of these two samples.

The degradation of the metal samples was assessed in SBF by measuring the weight loss to assess the degradation of Zn-based orthopedic implants in vivo. The results presented in Table 3 reveal that both WAAM-Zn and WR-Zn were degrading in SBF over time. The degradation rates were similar, with a marginally higher weight loss for WAAM-Zn.

**Figure 5:**
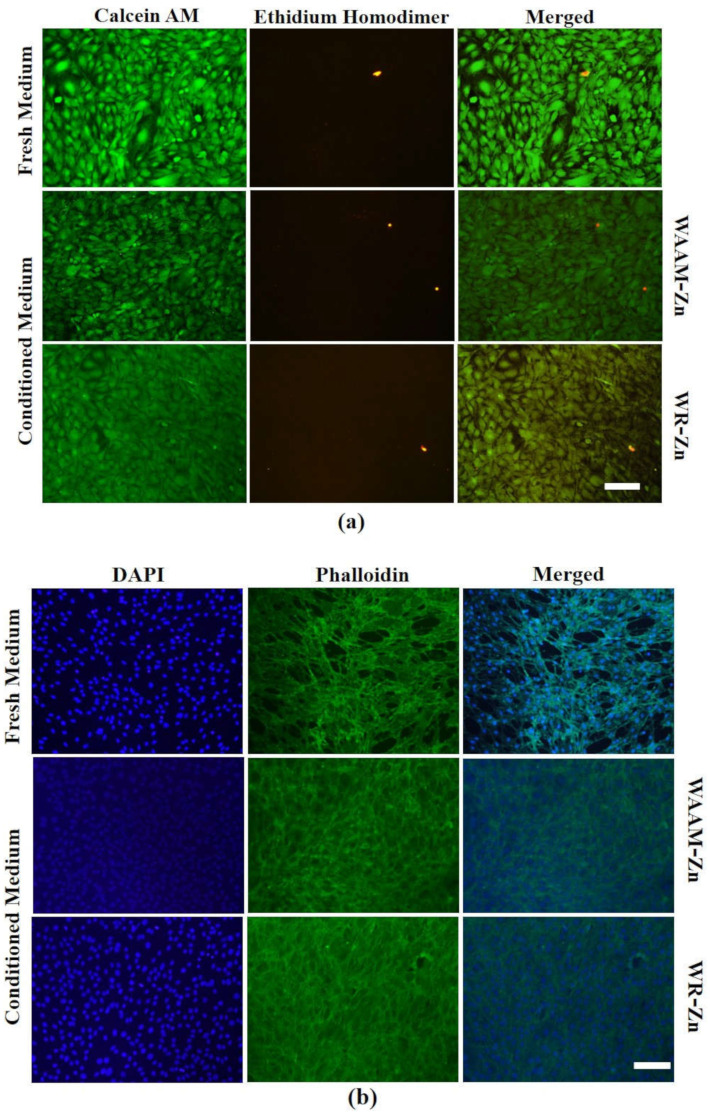
(**a**) Live/dead staining of MC3T3-E1 cells, performed after 48 h of treatment. (**b**) Representative images of nucleus and actin staining of treated cells. Fresh medium represents without treatment. (scale bar = 100 μm, applies to all figures).

**Figure 6:**
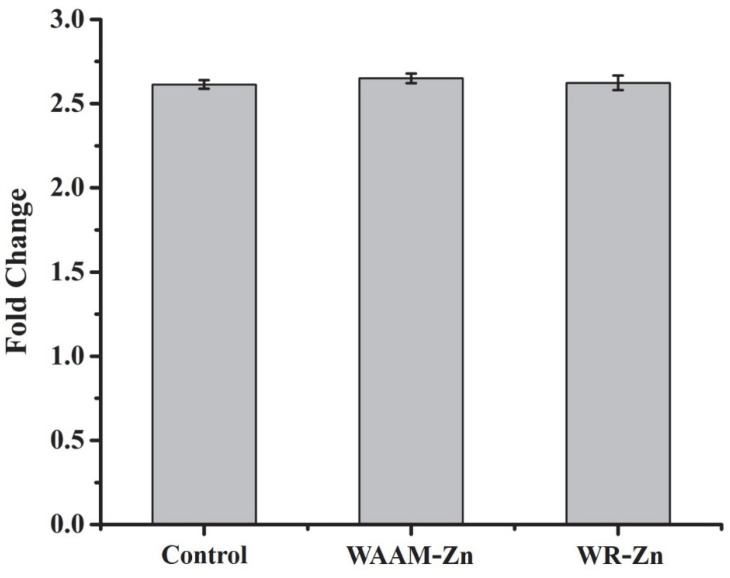
Fluorescence activity representation in fold change for WAAM-Zn and WR-Zn compared to control (fresh medium).

The cytocompatibility of WAAM-Zn was assessed in vitro by measuring the effect of any leaching of the initial degradation of the sample into the cell culture medium. The conditioned medium containing potentially cytotoxic components was prepared by the incubation of WAAM-Zn or WR-Zn in a complete cell culture medium for 24 h. MC3T3-E1 preosteoblasts were incubated in the conditioned medium for 48 h. The cell viability was assessed with live/dead staining and Alamar blue assay (Figure 5 and Figure 6). The fluorescent micrographs indicate no substantial loss of viability after exposure to the conditioned medium of WAAM-Zn or WR-Zn. The cell viability results determined by the Alamar blue assay are presented as fold changes for the control (fresh medium), WAAM-Zn, and WR-Zn before and after treatment with conditioned medium. The results indicate that there were no statistically significant differences between the control, WAAM-Zn, and WR-Zn. The cytotoxicity of degradable metals has been attributed to the alkalization of the cellular microenvironment owing to the rapid degradation of the alloys, which would result in the accumulation of hydrogen and metal ions [37]. The acute cell response measurements presented here indicate that WAAM-Zn is as cytocompatible as WR-Zn.

## 4. Conclusions

This work establishes the feasibility of fabricating dense Zn parts by wire-based AM for potentially engineering biomedical implants. The formation of voids due to the evaporation of Zn was minimized by the optimization of the parameters. The additively manufactured parts exhibited an equiaxial grain morphology and a size of 14 ± 5 μm, in contrast to 11 ± 4 μm for WR-Zn, which resulted in the marginally lower hardness of WAAM-Zn. The WAAM samples were heavily textured, with the directions <3 3 –6 2> or <0 0 0 2> of most of the grains aligned parallel to the building direction. The electrochemical response and degradation behavior of WAAM-Zn were similar to WR-Zn, with marginal differences. In vitro cell studies with MC3T3-E1 cells revealed that the excreted degradation products of both WAAM-Zn and WR-Zn showed cytocompatibility. This work demonstrates that the processing of Zn by WAAM is a promising route to leverage the advantage of fabricating near-net-shaped parts with mechanical, electrochemical, and biological performances that are similar to conventionally manufactured parts for biomedical applications. However, fabricating near-net-shaped implants with complex geometry through WAAM requires further modifications of the system, such as enabling the movement of the substrate. Further work is required to characterize the build parts for establishing WAAM as a means to prepare implants suitable for clinical use.

## Figures and Tables

**Table 1:** Process parameters for WAAM of Zn.

Parameter	Arc Power	Wire Feed Rate	Travel Speed	Standoff Distance
Values	400 W	160 mm/min	120 mm/min	5 mm

**Table 2:** Electrochemical parameters.

Sample	E_corr_ V	I_corr_ μA/cm^2^	Corrosion Rate mmpy	Microhardness HV0.3
WAAM-Zn	−1.18 ± 0.03	8.8 ± 1	0.45 ± 0.2	35 ± 2
WR-Zn	−1.13 ± 0.16	5.9 ± 0.8	0.3 ± 0.1	41 ± 1

**Table 3:** Biodegradation studies.

WAAM-Zn			WR-Zn		
Time	Sample Weight (mg)	Degradation Rate (mm/yr)	Time	Sample Weight (mg)	Degradation Rate (mm/yr)
Day 0	308.6	-	Day 0	291.6	-
Day 7	308.3	0.023	Day 7	290.8	0.063
Day 14	307.9	0.030	Day 14	290.3	0.040
Day 21	307.1	0.061	Day 21	289.8	0.040
